# PP84 Developing The Network For The Future Of Healthcare Through Telemedicine-Driven Diagnostic Innovation

**DOI:** 10.1017/S0266462324002538

**Published:** 2025-01-07

**Authors:** Pedro Galvan, José Ortellado, Ronald Rivas, Benicio Grossling, Enrique Hilario

## Abstract

**Introduction:**

The healthcare digital landscape has evolved and is crucial in shaping strategies for fortifying the health system, specifically in building a network for the future of health services. There is also considerable interest in digital health to facilitate innovation and access to health services in underserved hospitals. This study has evaluated the results of a telemedicine-driven diagnostic network in remote Paraguayan hospitals.

**Methods:**

This is a descriptive study, where the results using a digital telemedicine-driven diagnosis innovation in remote public hospitals were evaluated as a tool to improve equity and accessibility of specialized diagnostic services countrywide. For these purposes, the type and frequency of diagnosis performed through a digital telemedicine platform was determined.

**Results:**

During the study, a futuristic telemedicine-driven diagnostic innovation was implemented in 67 hospitals countrywide. The digital telediagnosis network facilitated tele-electrocardiography (ECG), teletomography, tele-electroencephalography (EEG), tele-Holter and tele-ABPM (ambulatory blood pressure monitoring). The implemented digital telemedicine network has performed 828,073 tests in total between 2013 and 2023. The most performed diagnoses were ECG (543,815 tests) followed by teletomography (266,750 tests), EEG (17,418 tests), Holter (43 tests), and ABPM (28 tests).

**Conclusions:**

According to our results, the telemedicine-driven diagnostic innovation network facilitates faster and more equitable access to tertiary-level diagnostic services for patients in remote underserved public hospitals in Paraguay. A widespread use-assessment is necessary before this platform is implemented.

